# Ultrasound-guided percutaneous drainage of infected pancreatic necrosis

**DOI:** 10.1007/s00464-013-3114-1

**Published:** 2013-08-13

**Authors:** Marek Wroński, Włodzimierz Cebulski, Dominika Karkocha, Maciej Słodkowski, Łukasz Wysocki, Mieczysław Jankowski, Ireneusz W. Krasnodębski

**Affiliations:** Department of General, Gastroenterological and Oncological Surgery, Medical University of Warsaw, ul. Banacha 1A, 02-097 Warsaw, Poland

To the Editor,

We appreciate the comments of Drs. Zerem and Omerović [1] in Letters to the Editor regarding our article. The authors suggest that necrosectomy in our series might represent overtreatment and that somewhat better results could have been achieved by insertion of additional catheters into the necrotic collections.

First, it should be emphasized that most data on percutaneous catheter drainage (PCD) come from retrospective studies based on low-level evidence and prone to selection bias. Consequently, the actual success rate might be lower than the 55.7 % reported by van Baal et al. [2] in their systematic review.

Percutaneous drainage in our series was successful for 33 % of the patients [3], similar to the success rate reported in a recent prospective study by van Santvoort et al. [4] (35 %). We think the lower success rate for PCD in our series was mainly due to a few technical complications that occurred early in our experience with this technique, and this issue is thoroughly explained in our article. The mortality rate of 17 % in our series is comparable with other data, which show a mortality rate of 5–29 % [2].

Before a decision is made to proceed with necrosectomy, we always considered improvement of percutaneous drainage by catheter upsizing or insertion of additional catheters to access poorly drained or undrained collections. The left retroperitoneal approach was preferably used in our series (13 of 18 patients) because it is the most dependable for gravitational drainage, allowing access also to the prepancreatic collections located in the lesser sac. Notably, nearly half of these patients had an additional percutaneous access, either transperitoneal or retroperitoneal.

On the other hand, subsequent ultrasound-guided interventions often are more difficult and sometimes impossible, whenever the necrotic collection has already been drained percutaneously. The liquid part of the necrotic collection is drained first, and the solid debris remaining within the necrotic cavity becomes hard to visualize sonographically because it appears iso- or hyperechoic to the adjacent tissues. Moreover, air that enters the necrotic collection causes artifacts and further obscures visualization of the collection. Therefore, insertion of additional catheters might be considered only in case of the undrained collections or when a catheter gets occluded and a sufficient amount of fluid re-accumulates. In the latter situation, however, it often is easier to upsize or exchange the catheter.

The aforementioned obstacles are inherent to ultrasound-guided procedures and can be overcome by using computed tomography for guidance. However, we did not use this technique at our institution.

In contrast to Drs. Zerem and Omerović [1], we believe that the size of the catheter matters and that its role in percutaneous drainage of infected pancreatic necrosis depends on the stage of the disease.

Our policy is to tailor the size of the catheter to the timing of PCD. We prefer to use the small-caliper catheters (9–14 Fr) for percutaneous drainage procedures performed within the first 4–6 weeks from the onset of disease. Next, we upsize the catheters if necessary. Thus, early in the course of acute pancreatitis, little necrotic debris can be drained. Therefore, the main role of the catheter is to drain the infected fluid rather than to remove the necrosis, and for this, both small- and large-caliper catheters might be equally successful. Later in the course of disease, the aim of the catheter is to evacuate more or less particulate debris, and large-bore catheters seem to be more effective for this purpose.

The principal aim of percutaneous treatment in infected pancreatic necrosis is to control the source of infection. A prolonged percutaneous drainage in septic patients who prove unresponsive to such management might cause a delay in appropriate treatment and result in mortality that might be potentially avoided by a timely surgical necrosectomy. Even currently in this era of minimally invasive techniques, open necrosectomy should not be regarded as overtreatment or failure of treatment, at least until more experience is gained based on well-designed prospective studies that allow us to know better the place of PCD in the management of infected pancreatic necrosis.
